# The Farther the Better: Effects of Multiple Environmental Variables on Reef Fish Assemblages along a Distance Gradient from River Influences

**DOI:** 10.1371/journal.pone.0166679

**Published:** 2016-12-01

**Authors:** Leonardo M. Neves, Tatiana P. Teixeira-Neves, Guilherme H. Pereira-Filho, Francisco G. Araújo

**Affiliations:** 1 Laboratório de Ecologia de Peixes, Departamento de Biologia Animal, Universidade Federal Rural do Rio de Janeiro, Campus Seropédica, RJ, Brazil; 2 Departamento de Ciências do Meio Ambiente, Universidade Federal Rural do Rio de Janeiro, Campus Três Rios, RJ, Brazil; 3 Laboratório de Ecologia e Conservação Marinha, Instituto do Mar, Universidade Federal de São Paulo, Campus Baixada Santista, Santos, SP, Brazil; Universita degli Studi di Genova, ITALY

## Abstract

The conservation and management of site-attached assemblages of coastal reefs are particularly challenging because of the tremendous environmental variation that exists at small spatial scales. In this sense, understanding the primary sources of variation in spatial patterns of the biota is fundamental for designing effective conservation policies. We investigated spatial variation in fish assemblages around the windward and leeward sides of coastal islands situated across a gradient of riverine influence (13 km in length). Specifically, relationships between rocky reef fish assemblages and benthic, topographic and physical predictors were assessed. We hypothesized that river induced disturbances may overcome local habitat features in modeling spatial patterns of fish distribution. Fish assemblages varied primarily due to the strong directional gradient of riverine influence (22.6% of the estimated components of variation), followed by topographic complexity (15%), wave exposure (9.9%), and benthic cover (8%). The trophic structure of fish assemblages changed from having a high abundance of invertebrate feeders in macroalgae-dominated reefs close to river mouths to a high proportion of herbivores, planktivores and invertebrate feeder species in reefs with large boulders covered by epilithic algal matrices, as the distance from rivers increased. This gradient led to an increase of 4.5-fold in fish richness and fish trophic group diversity, 11-fold in fish biomass and 10-fold in fish abundance. Our results have implications for the conservation and monitoring of assemblages patchily distributed at small spatial scales. The major role of distance from river influences on fish assemblages rather than benthic cover and topographic complexity suggest that managing land-based activities should be a conservation priority toward reef restoration.

## Introduction

One of the primary challenges of environmental studies is to determine the variables that influence the spatial distribution and structure of assemblages. Study of species-environmental relationships contributes to our understanding of the effect of ecological processes on distributions [1], our ability to predict responses of species to environmental change [2], and our ability to prioritize conservation goals [3,4]. In coastal reefs, the fundamental challenge for management of biological assemblages is that these habitats may be affected by multiple environmental variables [5]. Thus, understanding the primary sources of variation is necessary for the adoption of conservation strategies. In the case of reef fishes, these variables include benthic composition [6–8], topographic complexity [9,10], depth [9,11], wave exposure [12–14], and river discharges [15], among several others. All of these variables may interact to shape the abundance of individual species and the composition and structure of a fish assemblage.

Reef-fish assemblages are highly patchy, with considerable variation in abundance, diversity and biomass at small spatial scales (e.g., [5,16,17]). This patchiness is partly due to the sedentary nature of reef-fishes but also because the high intra and inter-habitat environmental variability restricts their movement [18,19]. For example, the increase in depth at relatively short distances from the shore provides additional surface area of habitat with a higher degree of structural diversity for marine life to utilize [20]. Coral growth and the complex substrate architecture provided by rocks create small-scale crevices that offer greater diversity and availability of shelter, nesting and foraging sites [21,22]. Therefore, higher topographic complexity increases site productivity and food availability, decreases competition and lead to a lower predator attack rate on individual preys (via a decrease in predator–prey encounters) and a lower interference rate (also via a decrease in predator–predator encounters) [23,24]. As a result, topographic complexity, species richness, diversity, total biomass, and abundance are positively correlated [10].

Wave exposure has been considered to be one of the key factors shaping reef fish assemblages, as the abundance of fast-swimming fish species with high-aspect-ratio fins has been found to be positively correlated with water flow and wave exposure by a number of studies in reefs around the world (e.g., [25–28]). High-energy, wave-driven environments also generate high abundances of specific food resources, such as zooplankton, and are positively correlated with the presence of planktivorous fish [14]. Despite numerous studies demonstrating that habitat structure and wave exposure are key factors influencing reef-fish assemblages, a comprehensive understanding of their behavior under gradients of environmental stress is still lacking, even for diverse tropical marine areas under high anthropogenic influences (e.g., [29,30]).

Environmental gradients in coastal reefs are commonly shaped by forces that vary with distance from a specific or combined source of stressors, such as land runoff (e.g., sediments, industrial and agricultural loads). Terrestrial loads not only significantly alter the structure and ecological function of biotic assemblages, but they also frequently result in altered biological diversity and productivity [31]. In coastal reefs, environmental variables related to continental proximity (i.e., siltation, water clarity, nutrient levels) drastically alter benthic composition as exposure to river influences increases [31,32]. Turf-forming algae are generally associated with highly disturbed habitats, whereas zoanthids and massive corals dominate less disturbed areas (e.g., [33–35]). Yet, measuring the supply of terrestrial sediments to the nearshore zone gives little insight on reef processes because sediment flux differ with local tidal regimes, different levels of exposure to waves or currents, and local bathymetry [36,37]. However, to account for anthropogenic influences on fish distributions, simple distance metrics have been used as reasonable proxies for the intensity of sedimentation from deforestation and subsequent land-use practices [4].

The majority of studies that have investigated patterns of fish distribution across riverine gradients [15,38] have focused on coral-dominated reefs of the Caribbean and the Pacific. In contrast, little to no information is available for tropical, algal-dominated rocky reefs. On the tropical-subtropical transition zone of the Brazilian coast, rocky shores represent the primary habitat for reef fishes and reef-associated biota (e.g., [39]). In the Brazilian insular complex of the Ilha Grande Bay, islands are distributed across a gradient of distance from rivers and therefore differ substantially in their environmental conditions [40]. Islands close to the river mouths are characterized by extremes in sedimentation and turbidity after flood events; in fact, the substrate may be buried by sediments. In contrast, islands far from the river influences have clearer waters, with an abundance of lower suspension sediments, in addition to a greater availability of hard substrate. Addressing the complex multivariate features of biodiversity in coastal areas, by accounting for co-occurring disturbances and natural variation in environmental conditions is critical [41]. In this study, we aimed to identify the most influential environmental variables on the spatial patterns of a reef fish assemblage of coastal islands. Specifically, we examined the relative importance of benthic (i.e., percentage cover of morpho-functional groups) topographical (i.e., boulder size, number of holes, crevices) and physical variables (i.e., distance from rivers and wave exposure) on fish assemblages. In addition, we determined which species were driving these multivariate effects. We hypothesized that the expected role of habitat complexity in increasing fish richness and diversity is minimized in reefs under a strong river influences, as the ability of marine sessile organism and fish larvae will depend on suitable settlement substrates. The benefits of examining changes in fish assemblages across defined environmental gradients include understanding which assemblage parameters are more heavily influenced by both discrete and extreme changes in habitat conditions. This knowledge will be useful in determining how environmental changes related to both natural and anthropogenic disturbances may affect rocky reef fish assemblages. This study also promises to facilitate reef managers and conservation planners to incorporate key variables driving fish composition and diversity in their decisions about management tools and location of reserve sites.

## Materials and Methods

### Study area

**Fig 1:**
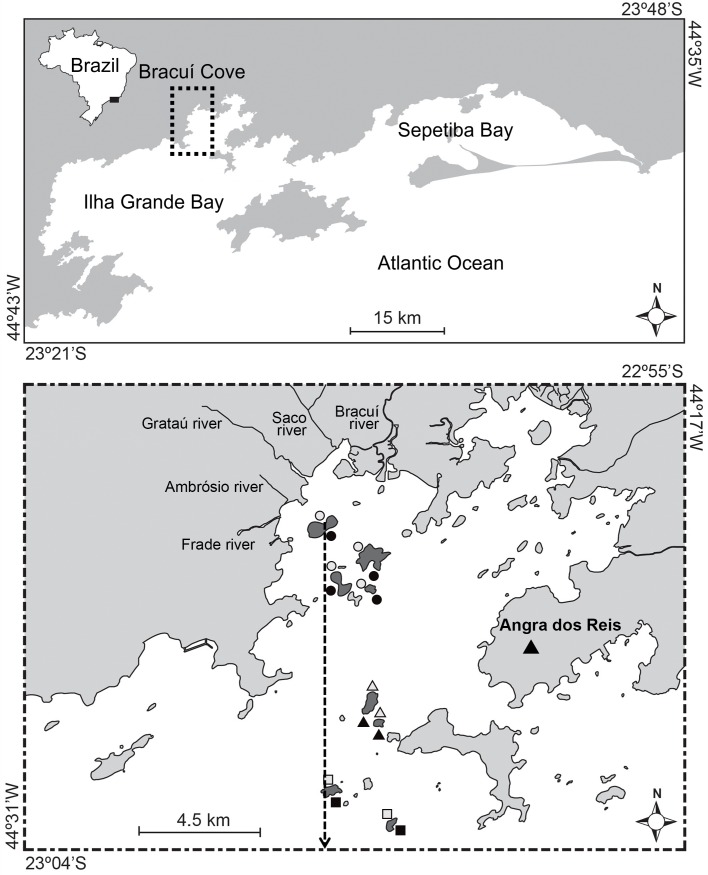
Map of the study area showing the sampling sites. The arrow indicates the studied gradient, with sites allocated less than 5 km (circles), from 8 to 10 km (triangles) and more than 11 km (squares) of distance from the river confluence area. Wave exposure (sheltered, empty symbols; exposed, dark symbols).

This study was conducted along insular rocky reefs between 2 and 8 m deep on the Ilha Grande Bay (23°06 S, 44°42 W), southeastern Brazil, during the summers of 2011 and 2012 (Fig 1). Ilha Grande Bay covers an area of approximately 1,000 km^2^ and contains approximately 350 islands surrounded by shallow water (typically no more than 8 m in depth), several of them forming granitic rocky shores [42]. Averaged accumulated annual rainfall is 1770 mm, ranging from 180 mm during the dry⁄winter season (June–August) to 750 mm during the wet⁄summer season (January–March). Temporal changes in rainfall and in river flow result in two seasons of comparatively low (winter) and high (summer) river influence, and two intermediate seasons (spring and autumn). Mean water temperature ranges from 20°C to 31°C, while salinity ranges from 29 to 36 [43]. Local water masses are influenced by winds and tides with a mean amplitude of 1.6 m [44].

Ilha Grande Bay has a heavily indented shoreline and coastal mountains reach the coastline, leaving little space for the formation of a coastal plain [45]. At the northern region of the bay, the Bracuí Cove receives several small freshwater inputs from the rivers Frade, Ambrósio, Grataú, Saco and Bracuí. These rivers have watershed areas ranging from 16 km^2^ to 190 km^2^ [46] and drain into a confluence area in the Bracuí Cove, creating an area subject to inputs of terrestrial sediment and land-based activities. This confluence area was considered to be the starting point of the gradient of decreasing exposure to river influences in this study (Fig 1). Some villages of the Angra dos Reis city are distributed around these rivers and strongly impacted the rivers due to channelization and dredging for shipping, construction and the removal of mangrove forests in the coastal area. Despite these impacts, sediments in the coastal area were not considered to be polluted by metals according to [40]. Economic activities conducted in the bay include tourism, power generation (thermonuclear power plants), shipyards, private marinas, oil terminals and fisheries, as well as a commercial port, all of which indirectly influence the study area [29,40].

Sixteen sampling sites were allocated along a gradient of distance from the river confluence area, encompassing eight coastal islands (Fig 1). At each island, two sites were surveyed according to wave exposure, one sheltered and the other exposed to wave action. Exposed sites are most subjected to stronger swells and winds from the southwest, whereas protected locations lie on the protected side of each island (Fig 1). Sites close to the river confluence (< 5 km) are most exposed to riverine inputs and human-induced disturbances, whereas those far (> 10 km) are less exposed to river discharges and to sediment deposition. There is no relevant island runoff over adjacent reefs due to the absence of insular rivers and eventual sediment is trapped by the forest cover. High rainfall (20 to 60 mm/3 h) during the summer creates large plumes of suspended solids at reefs close to rivers (S1 Fig.; LMN and TN, personal observation). Thus, sediment loads on the rocky reef appear to be an important regional disturbance.

### Sampling surveys

Underwater visual censuses were performed by scuba diving along transects 20 m long and 2 m wide (40 m^2^) in order to account for the lowest levels of visibility at the study sites [13,47]. At each site, 9–27 transects were randomly sampled, totaling 252 transects (first summer, 114; second summer, 138 samples; S2 Table). Sample size varied between sites because of inclement weather. Fish transect surveys were performed twice. During the first survey, the observer noted conspicuous species; during the second survey, the observer focused on searching beneath rocks and crevices to detect cryptic species. The sampling unit, number of fish per transect, was defined as the pooled number of conspicuous and cryptic species. Samples were performed under stable oceanographic conditions, between 9:00 and 14:00 h, during neap tide, near quarter moon.

We used high resolution digital images to quantify benthic communities and the topographic complexity of the sea-bottom at the study sites (Fig 1). A housed digital camera was mounted on a 0.36-m^2^ polyvinyl chloride (PVC) photo quadrat framer. A total of 30 photographs were taken at each site during each summer, totaling 60 foto-quadrats. Photographs were taken randomly along the same fish transect line. Topographic complexity variables measured on rocky reefs consisted of two scales: i) small-scale complexity, that was considered to be the number of holes and crevices in each quadrat, and ii) large-scale complexity, that was considered to be the size of rocky boulders. Small-scale complexity was measured by counting the total number of holes and crevices (gaps between structures that could provide a path for a fish to escape a predator) from each photograph. Although holes and crevices of different sizes were recorded (<30 cm; 30 cm–1 m; >1 m, according to Aburto-Opereza and Balart [48]), we combined all size categories into a single measure called ‘number of refuges’. At the same position from which each photo was taken, we estimated the height in meters of the tallest rocky boulder (boulder size).

Images were also used to measure the percentage of benthic cover using Coral Point Count with Excel Extensions software–CPCe 3.4 [49] by overlaying 20 random points on each image and identifying the substratum under each point. Benthic sessile and semi-sessile organisms, expressed as percentage of benthic cover, were grouped into the following thirteen categories (adapted from [33]): hard coral, coenocytic thalli (e.g., macroalgae from the *Caulerpa* genus), crinoidea, crustose coralline algae, tunicate, epilithic algal matrix (EAM, i.e., aggregate with less than cm high of filamentous algae), echinodermata, fleshy algae, hydrozoa, octocoral, sessile polychaeta (e.g., phragmatopoma), soft coral and sponge.

Wave exposure was categorized as high versus low exposure (i.e., exposed or windward versus protected or leeward shores) around each of the eight islands. We accounted for potential river influences on fish distributions by measuring the distance of a reef from the confluence of local rivers into the coastal area (hereafter called river confluence area).

This research was conducted under SISBIO Collection of Species Permit number 10707 issued by ICMBio, Brazilian Environmental Agency.

### Data analysis

The following fish assemblage parameters were used: fish assemblage structure, fish richness, fish abundance, fish biomass and fish trophic group diversity. Total numbers of species (richness) and individuals (abundance) were calculated based on observations from each transect. Fish biomass was estimated by length-weight relationships: W = a∙Lb where parameters a and b are the parameters of the allometric growth equation [50]. FishBase and additional literature [51–53] were used as sources of this information (S1 Table). When coefficient values were not recorded for a species, we used coefficients for the closest related species or genera. Fish taxa were grouped into seven trophic groups based on the literature [13,54,55]: mobile invertebrate feeders, sessile invertebrate feeders, carnivores, omnivores, planktivores, roving herbivores and territorial herbivores. Fish trophic group diversity was then calculated using the Shannon-Weiner diversity index, H’, which takes into account both abundance and the number of trophic groups.

The effects of the environmental predictors (distance from the river confluence area, boulder size, number of refuges, benthic cover and wave exposure) on fish assemblage parameters were assessed by considering site as the lowest level of replication. A single value for each site in each year was calculated (average) for the continuous predictors (covariates) and for the response variables. Average values of the predictors at each site were used in the analysis, except for benthic cover. A distance-based principal coordinate analysis (PCO) on the benthic cover data was performed to combine all substrate categories (13 variables, see previous section) into a single variable using the first PCO axis scores as a covariate. Variability in benthic cover among reefs was investigated by plotting PCO1 scores against dominant substrate categories. The existence of highly correlated predictor variables and the need for data transformation was assessed using a draftsman plot. Pairwise correlation coefficients were calculated between all covariates (distance from the river confluence area, boulder size, number of refuges and PCO1 scores of the benthic cover data) and none of these covariates displayed any collinearity (r <0.7; [56]). As the covariates had a low degree of skewness, raw data were used for the analysis following [57]. The data were then organized into six matrices: five matrices corresponding to each response variable (fish assemblage, fish richness, abundance, biomass, fish trophic group diversity) and one covariate data matrix with the distance from the confluence area, benthic cover (first PCO1 axis), number of refuges and boulder size.

We used permutational multivariate analysis of variance (PERMANOVA; [58]) with a Type I (sequential) sum of squares to calculate p-values, where fish assemblage, fish richness, fish abundance, fish biomass and fish trophic group diversity (H’) were the response variables, and distance from the river confluence area, boulder size, number of refuges and benthic cover (first PCO axis) were covariates. Wave exposure (2 levels, exposed versus sheltered locations) and sampling year (2 levels, summer of 2011 and 2012) were fixed and random factors, respectively. Sampling year was included in the model because each site was repeatedly sampled over time. When a factor (main effect or interaction) in the model was not significant, the p-value was higher than .25 and the proportion of variability explained by the factor lower than 5%; we removed the factor from the analysis, and the model was rerun without the excluded factor following [59].

The relationship between the covariates and the fish assemblage structure was investigated using distance-based redundancy analysis (dbRDA, [60,61]). Pearson correlations with the first two dbRDA axes were examined to identify the dominant species driving the fish assemblage response to habitat and physical variables. The univariate response variables (fish richness, fish abundance, fish biomass and fish trophic group diversity) were regressed against the covariates with significant effect according to PERMANOVA to define the nature of the relationship (positive or negative).

To investigate the effect of wave exposure and groups of sites with similar assemblage compositions on the abundance of selected species (frequency of occurrence > 40% and Pearson correlation with the dbRDA axis > 0.3), we built a new PERMANOVA. Sites were assigned qualitatively to groups based on their distribution along the first 2 axes of the dbRDA (3 levels, corresponding to the 3 groups of sites according to the dbRDA, fixed factor), according to wave exposure (2 levels, fixed factor) and sampling year (2 levels, random factor). PERMANOVA pairwise comparisons were performed to assess differences in fish abundance between exposed and sheltered sites and groups. We used the total number of samples (252) for these analyses. Prior to analysis, fish assemblage, fish richness, abundance, biomass and fish trophic group diversity data were square root transformed. Bray-Curtis similarity matrices were calculated for multivariate data while Euclidean similarity matrices were calculated for univariate variables.

## Results

### Benthic cover and topographic complexity

Benthic cover surveys revealed that the rocky reefs were dominated by epilithic algae matrix (EAM), soft coral and fleshy algae, which together accounted for more than 90% of the total substratum cover. Mean (±SE) EAM cover ranged from 17.7% (±2.7) to 95.2% (±1.0), fleshy algae cover from 2.5% (± 0.6) to 48.8% (±2.3) and soft coral cover from 0 to 69.4% (±3.4) per reef (S2 Table). The mean (±SE) number of refuges varied from 0.37 (±0.1) to 4.11 (±0.3), whereas boulder size ranged from 29.8 cm (±0.6) to 119 cm (±13.3). Sites exposed to wave action usually had larger boulder sizes than sheltered sites (S2 Table).

### Fish composition

A total of 13,027 individuals from 78 fish taxa (mostly to species level) were recorded. *Eucinostomus* spp, *Coryphopterus* spp. and *Kyphosus* spp. were not identified at species level due to the difficulty of the specific determination by direct visual observations. The 10 most abundant species accounted for approximately 86% of all fish recorded in this study. Six of these 10 species also had the highest biomass. The mean species richness per transect was 7.7 (±0.2) species, with a minimum of 2 and a maximum of 18 species. The mean number of individuals per transect was 52.2 (±2.8), with a minimum of 5 and a maximum of 257 individuals. The mean fish biomass per transect was 2.6 (±0.2) kg, and ranged from 0.2 kg to 15.7 kg (mean ±SE for all variables). Some species occurred either exclusively or predominantly in certain distances from the river confluence area. For example, *Haemulon steindachneri*, *Serranus flaviventris* were very abundant in the 8 sites from 1 to 4.5 km of distance, whereas only 1 individual of *H*. *steindachneri* was observed at the 4 locations from 11 to 13 Km. In contrast, the reverse trend was exhibited by *Sparisoma frondosum*, *Pempheris schomburgkii*, while species such as *Stegastes fuscus* and *Haemulon aurolineatum* were regularly present (occurrence > 70%) in sites from 8 to 13.1 km.

### Influences of predictors variables on fish assemblage structure

The distance from the river confluence area was the best predictor of spatial changes in fish assemblages (22.6% of total variance), followed by wave exposure (9.9%), benthic cover (8.0%), distance and boulder size interaction (7.9%), number of refuges (7.8%) and boulder size (7.3%). There was also a significant effect of sampling year (the two summers) that explained 7.3% of the variance (Table 1).

**Table 1:** Results of PERMANOVA testing for differences in fish assemblage structure, in response to distance from the river confluence area, boulder size, number of refuges, benthic cover (covariates), exposure (fixed factor) and year (random factor) and interaction effects.

Source	df	SS	MS	ECV	Pseudo-F	P
Distance	1	24215	24215	22.6	38.9	***
Refuges	1	3330.7	3330.7	7.8	5.4	***
Boulder size	1	1472.9	1472.9	7.3	2.4	***
Benthic cover	1	1350.2	1350.2	8.0	2.2	**
Distance*Refuges	1	1003.8	1003.8	4.2	1.6	ns
Distance*Boulder size	1	1967.9	1967.9	7.9	3.2	***
Boulder size*Refuges	1	991.03	991.03	4.8	1.6	ns
Wave exposure	1	1179.9	1179.9	9.9	1.9	*
Year	1	1843.4	1843.4	7.3	2.9	***
Residuals	22	13663	621.06			
Total	31	51018				

(df = degrees of freedom, SS = sum of squares, MS = mean sum of squares, ECV = percent estimated components of variation, F = pseudo-F

* = p<0.05

** = p<0.01

***p<0.001

ns = non-significant).

**Fig 2:**
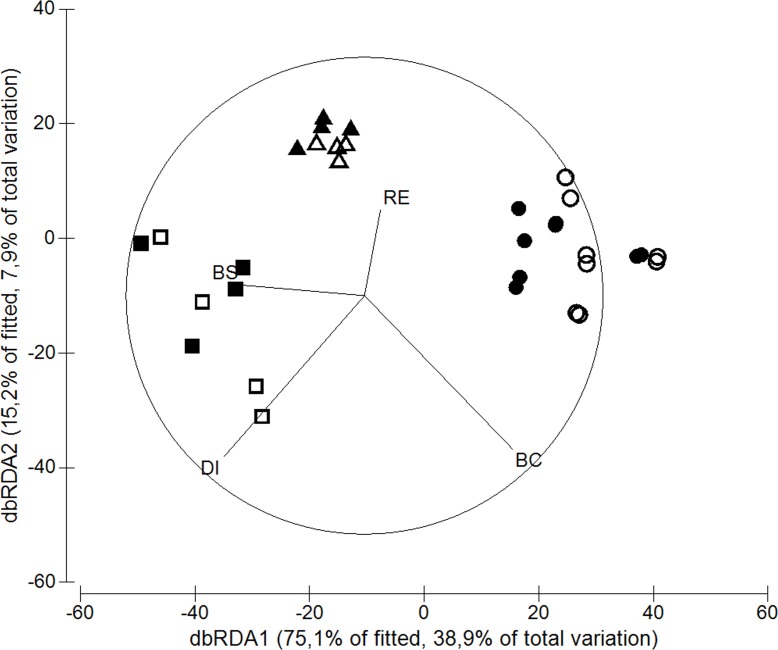
Distance-based redundancy analysis (dbRDA) demonstrating the relationships between fish assemblage structure and the covariates. DI, distance from the river confluence area; BS, boulder size; BC, benthic cover (PCO1 axis); RE, number of refuges. Highest BC values represent reefs dominated by EAM, while lower values represent soft coral dominated-reefs. Sampling sites were indicated according to the proximity of the river confluence area (circle, close; triangle, intermediate; square, far) and degree of wave exposure (sheltered, empty symbols; exposed, dark symbols).

**Fig 3:**
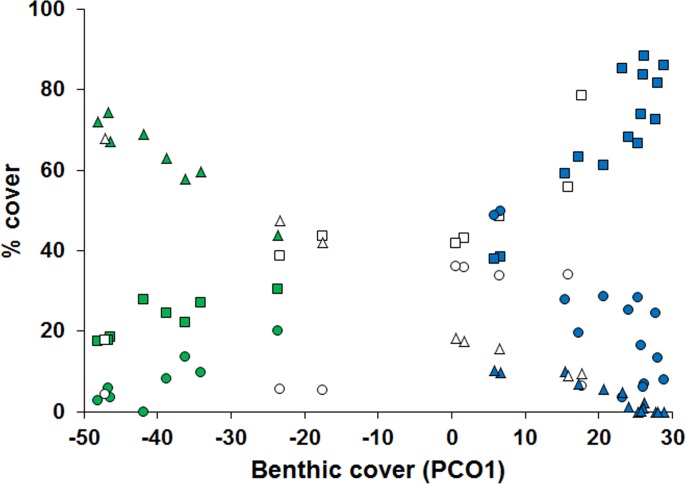
Relationships between EAM, fleshy algae and soft coral cover with the first PCO axis. Squares–EAM, circles–fleshy algae, triangles–soft coral. Colors represent the three groups based on the distribution of the sites along the first 2 axes of the dbRDA: Blue, Close reefs, located from 1.4 to 4.5 km; green–Intermediate reefs, located from 8 to 9 km and white–Far reefs, located from 11.2 to 13.1 km.

We found a strong relationship between fish assemblages and covariates (Fig 2). The first distance based redundancy analysis (dbRDA) axis accounted for 38.9% of the total variation in fish assemblages and distinguished sites far from river influences with higher boulder sizes and generally less dominated by a specific benthic cover type (PCO1 scores from –25 to 10; Fig 3) from sites close to this influence, dominated by EAM (PCO1 scores from 15 to 30). The second dbRDA axis accounted for 7.9% of the variation in fish assemblages and accounted for sites at intermediate distances from the river confluence area, dominated by soft coral (PCO1 from –50 to –30) and with higher number of refuges from sites far from the rivers, dominated by EAM (Figs 2 and 3).

A large number of species representing 7 trophic groups were negatively correlated with Axis 1, indicating that they were associated with large distances from the river confluence area and large boulders sizes (r > 0.4; S3 Table). These species by trophic group were the sessile invertebrate feeders *Chaetodon striatus* and *Canthigaster figueiredoi*, the mobile invertebrates feeders *Halichoeres poeyi*, *Holocentrus adscensionis* and *H*. *aurolineatum*, the omnivorous *Abudefduf saxatilis*, *Pomacanthus paru* and *Parablennius marmoreus*, the roving herbivores *Sparisoma frondosum* and *Acanthurus chirurgus*, the territorial herbivores *S*. *fuscus*, the planktivore *Chromis multilineata* and the carnivore *Epinephelus marginatus*. The mobile invertebrates feeders *Labrisomus nuchipinnis*, *Pareques acuminatus* and *Malacoctenus delalandii*, the territorial herbivores *Stegastes variabilis* and the roving herbivores *Sparisoma axillare* and *S*. *frondosum* were negatively correlated with axis 2, indicating that they were associated with the largest distances from river influences and PCO1 scores (benthic cover dominated by macroalgae). The invertebrate feeders *H*. *steindachneri*, *S*. *flaviventris* and *Sphoeroides greeleyi* were positively correlated with axis 1, indicating that they were associated with proximity to rivers and positively associated with PCO1. The positive correlations (> 0.3) of the territorial herbivores *S*. *fuscus*, the omnivore *Coryphopterus* spp, the mobile invertebrate feeder *Emblemariopsis signifer* and the herbivore *Scartella cristata* with axis 2, indicated that they were associated with a higher number of refuges (S3 Table).

### Spatial patterns of selected species

**Fig 4:**
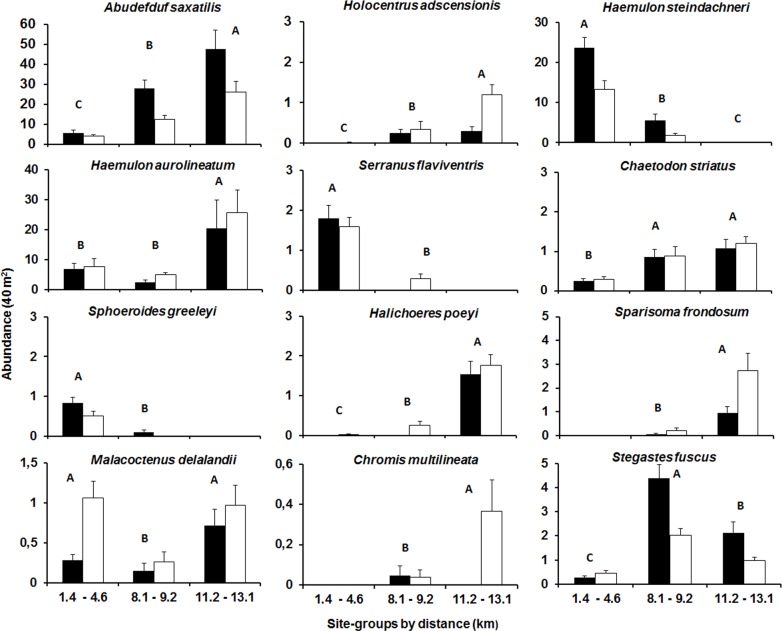
Mean abundance (individuals per 40 m^2^±SE) of selected species. Three site-groups were defined according to the distribution of the sites along the dbRDA axis. Black and white columns represent sheltered and exposed areas to wave exposure, respectively. Capital letters show pairwise results from PERMANOVA for the three groups. Significant results of pairwise comparisons for wave exposure (p < 0.001): *Abudefduf saxatilis* = sheltered > exposed; *Holocentrus adscensionis* = exposed > sheltered; *Malacoctenus delalandii* = exposed > sheltered; *Sparisoma frondosum* = exposed > sheltered; *Chromis multilineata* = exposed > sheltered; *Stegastes fuscus* = sheltered > exposed.

The abundance of several taxa differed significantly among the three dbRDA groups which corresponded to the gradient of distance from the river confluence area (close, intermediate and far; S4 Table) and to a lesser extent between the degree of wave exposure (sheltered and exposed), see S5 Table and Fig 4. Reefs close to the river confluence were characterized by large EAM cover (Table 2). In the terms of fish composition, these sites were very different from reefs far from river mouths with the highest boulder sizes. Close reefs have the highest abundances of *H*. *steindachneri*, *S*. *flaviventris* and *S*. *greeleyi*, while far reefs were characterized by *H*. *adscensionis*, *C*. *striatus*, *H*. *poeyi*, *S*. *frondosum and C*. *multilineata*. Intermediate reefs were dominated by Soft Coral cover and the highest number of refuges (Table 2). At these sites, *Stegastes fuscus* was more abundant, while *M*. *delalandii* showed the lowest abundance. *Abudefduf saxatilis* and *S*. *fuscus* were more abundant at sheltered areas, while *H*. *adscensionis*, *M*. *delalandii*, *S*. *frondosum* and *C*. *multilineata* were more abundant at exposed areas. Differences in the abundance of *A*. *saxatilis*, *H*. *steindachneri*, *C*. *striatus*, *H*. *poeyi*, *S*. *frondosum*, *S*. *fuscus* and *M*. *delalandii* were detected between sampling years (S5 Table).

**Table 2:** Key environmental predictors (mean ± SE) by site-groups.

Site-groups		Close		Intermediate		Far	
Predictors		Sheltered	Exposed	Sheltered	Exposed	Sheltered	Exposed
Distance from the river confluence area		3.1±1.2	3.1±1.2	8.5±0.6	8.9±0.3	12±1.1	12.3±1.3
Benthic cover	EAM	80.9±1.1	64.1±1.6	20.3±2.0	28.4±2.7	37.6±2.4	56.3±3.1
	Fleshy Algae	11.6±0.9	21.4±1.2	5.5±1.1	12.3±2.3	13.7±1.6	23.3±2.5
	Soft Coral	2.7±0.5	3.1±0.7	68.3±2.7	54.6±3.3	41.5±2.9	15.5±2.7
Boulder size		39.9±0.8	56.6±2.3	47.0±1.8	76.8±5.1	57.9±2.0	112.4±8.7
Number of refuges		2.1±0.1	1.0±0.1	3.9±0.2	3.0±0.2	2.5±0.2	0.6±0.1

### Relationships between the predictors and the univariate fish parameters

**Fig 5:**
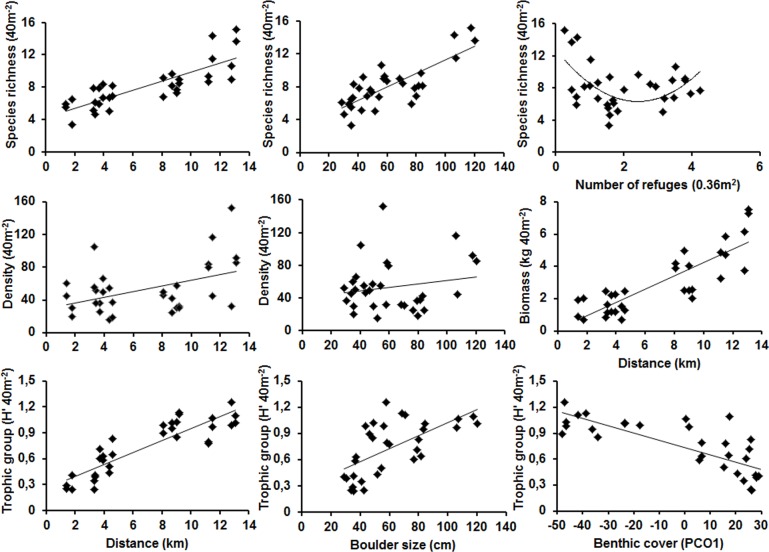
Relationships between physical, topographic and benthic predictors and fish assemblage parameters. Scatter plots of the covariates that had a significant effect on fish richness, abundance, biomass and fish trophic group diversity according to PERMANOVA.

Fish richness was positively influenced (p<0.001) by the combined effect of increased distance from the river confluence area that explained the largest component of variance (ECV = 35.9%), greater topographic complexity at a large scale (> boulder size; ECV = 10.7%) and lower complexity at a small scale (< number of refuges; ECV = 14.2) (Table 3, Fig 5, S6 Table). Fish trophic group diversity was also strongly influenced by a positive relationship with distance from the river confluence (ECV = 31.4%) and increased boulder size (ECV = 13.2%). Trophic group diversity was the only fish parameter influenced by benthic cover (ECV = 19.2%), with a more complex trophic structure related to greater EAM cover instead of Soft Coral cover (Table 3, Fig 5, S6 Table). For fish abundance, boulder size was the most influential predictor (ECV = 17.4%), although distance still had a significant positive effect (ECV = 12.4). On the other hand, fish biomass was predicted only by distance from the river confluence area (ECV = 38.2). There were no significant interactions between the factors and the covariates or between the covariates for most fish parameters. An exception was observed for fish trophic group diversity, which showed significant interactions between the distance from the river confluence and topographic complexity measures, and between benthic cover and boulder size (Table 3).

**Table 3:** PERMANOVA results based on Euclidian distance measures for a. fish richness, b. abundance, c. Biomass and d. Fish trophic group diversity.

	a. Fish richness					b. Fish abundance					c. Biomass					d.Trophic group diversity				
Source	*df*	MS	ECV	F	*P*	*df*	MS	ECV	F	*P*	*df*	MS	ECV	F	*P*	*df*	MS	ECV	F	*P*
Distance	1	4.5	35.9	172.0	***	1	18.9	12.4	13.3	***	1	10.0	38.2	123.9	***	1	2.3	31.4	394.2	***
Refuges	1	0.7	14.2	26.5	***	1	4.2	5.0	2.9	ns	-	-	-	-	-	1	0.3	3.2	5.0	ns
Boulder size	1	0.2	10.7	6.2	**	1	13.5	17.4	9.5	***	1	0.2	4.6	2.6	ns	1	0.1	13.2	24.9	***
Benthic cover	-	-	-	-	-	1	4.8	10.9	3.4	ns	1	0.3	10.2	3.3	ns	1	0.2	19.2	37.6	***
Distance*Boulder	1	0.1	5.5	4.2	ns	1	3.4	4.6	2.4	ns	-	-	-	-	-	1	0.06	5.2	9.7	*
Distance*Refuges	-	-	-	-	-	-	-	-	-	-	-	-	-	-	-	1	0.03	5.5	4.4	*
Benthic cover*Boulder	-	-	-	-	-	-	-	-	-	-	-	-	-	-	-	1	0.03	5.4	5.5	*
Wave exposure	-	-	-	-	-	1	3.4	10.1	2.4	ns	1	0.1	6.1	1.8	ns	1	0.03	7.8	4.8	ns
Year	1	0.6	18.5	23.7	***	1	24.3	20.1	17.2	***	1	1.7	21.8	21.0	***	-	-	-	-	-
Residuals	26	0.03				26	1.4				26	0.1				23	0.01			
Total	31					31					31					31				

(df = degrees of freedom, MS = mean sum of squares, ECV = percent estimated components of variation, F = pseudo-F

* = p<0.05

** = p<0.01

***p<0.001

ns = non-significant,—factor, covariate or interaction that were not significant, had p value was higher than .25 and the proportion of variability explained lower than 5%).

Despite these significant influences of the predictors, sampling year explained the largest component of variance for fish biomass and fish abundance (21.8% and 20.1%, respectively) and was the second most important predictor of fish richness (18.5%) (Table 3). However, interactions with sampling year were not significant, indicating that the environment-species relationships were consistent over the two sampled summers.

## Discussion

This study provides the first attempt to disentangle the drivers of rocky reef fish assemblage variation along a gradient of river influences in the South Atlantic. The relative importance of habitat structure and physical disturbances (or proxies, such as distance from rivers) on fish dynamics was modelled without a priori classification of sites into groups. Previous studies in this region have pre-categorized sites [13,17,20], which may produce misleading results considering the patchy nature of coastal reefs. In this study, habitat structure varied greatly among small spatial scales. Reefs that are close to each other can have very distinct dominant benthic cover and topographic complexity (S2 Table). For example, soft coral cover increased from 7.7 to 70% and boulder size varied from 36 to 80 cm between reefs spaced only 4 km apart. Overall, reefs were characterized by few benthic groups, namely EAM, fleshy algae and soft coral, with rocky boulders being the main provider of topographic complexity in the area.

### Habitat structure and physical influences on fish assemblages

Distance from river influences was the best predictor of spatial changes in fish assemblages. This ‘distance effect’ was particularly related to fish biomass, which alone explained 38.2% of the variance. However, for fish assemblage structure, fish richness, fish abundance and fish trophic group diversity, the combined effects of local habitat features (topographic complexity and/or benthic cover) and the gradient of distance from riverine (land-based) influences were associated with assemblages that were highly heterogeneous at small spatial scales. Distances from sources of disturbances (e.g. river mouths and reef channels) have been shown to play important roles in structuring fish assemblages by mediating the availability of shelter and food resources [4,14,15,62] as well as increased river runoff could reduce settlement success of coral and fish larvae [31,63].

Three distinct fish assemblage structures and the factors that explained their variance were identified. The first group (“close reefs”, see results) included invertebrate fish feeders *Haemulon steindachneri*, *Serranus flaviventris* and *Sphoeroides greeleyi*, which were more abundant in macroalgal-dominated reefs (mainly fleshy algae and EAM) with low levels of large-scale topographic complexity and a higher degree of riverine influence. Macroalgae are known to harbor more abundant and diverse assemblages of invertebrates because they provide a greater availability of surface for colonization by fauna and epiphytic algae and provide more food for benthic invertivores [64,65]. The second group (“intermediate reefs”) included small-sized cryptobenthic species (e.g., *Coryphopterus* spp., *Scartella cristata* and *Stegastes fuscus*) that were more abundant at higher small-scale topographic complexity reefs (> number of refuges) dominated by soft coral at intermediate distances from the river confluence. The abundance of refuges is especially important for small reef fishes for mitigating normally high rates of predation [66,67]. On the other hand, small-bodied predators that are capable of maneuvering within structured areas may benefit from foraging in a microhabitat with a high degree of prey availability [68]. [69] also found a greater abundance of *S*. *fuscus* in areas with high quantities of holes, which they use as shelter [47]. Finally, the third group (“far reefs”) had a more even distribution of trophic classes such as herbivores (*Sparisoma frondosum*), planktivores (*Chromis multilineata*) and also invertebrate feeders (*Halichoeres poeyi* and *Holocentrus adscensionis*) which were associated with reefs of a higher degree of large-scale topographic complexity (> boulder size) that are generally less dominated by a specific benthic cover type at the end of the sampled gradient. Herbivores were associated with a high availability of food resources (EAM) present in large boulders. There is some evidence that tall structures (e.g., coral colonies) increased vigilance of approaching predators [70] that may be particularly beneficial for planktivores as their food is more abundant higher in water column [71]. In these reefs, herbivory is probably not heavily impaired by sediment deposition [72–75] and foraging success of planktivorous fishes is not reduced by high levels of suspended sediment concentrations [63,76].

We found remarkable variation in the abundance of roving herbivorous fishes, that were absent in reefs close to the river confluence (< 5km, see S4 Table). The availability of algae in itself was not the reason for a decrease in herbivore density as proximity to river mouths increases because EAM was the dominant benthic cover (average % cover > 80.9±1.1 SE) in reefs experiencing high levels of river discharge (< 5 km from the river-influenced area). Sediment in algal turfs has been shown to suppress herbivory by coral reef fishes, with experimentally reduced sediment loads resulting in higher herbivore feeding rates [74,75]. However, differences in the species composition of EAM may be related to observed patterns of herbivore distribution. EAM have been grouped into a morpho-functional group [33] and only recently have detailed data showed that they are more variable than originally expected [77]. We expected that EAM composition depends on the degree of riverine influence; thus, epilithic algae-forming species composition may influence fish assemblages in different ways. Because of the importance of EAM for herbivorous fishes [78,79], study of their composition and functional role along environmental gradients are important future avenues of research in reef ecology.

There is little evidence to suggest that wave action had large effects on fish parameters, which was marginally significant, explaining 9.9% of the variation in fish assemblage structure. This effect may be in part due to the relatively small difference between ‘exposed’ and ‘sheltered’ sites of islands located in an enclosed sea with a relatively short fetch, which agrees with the findings of [80]. Few species appeared to be directly influenced by the motion of the water in this study. An example is the mid-water schooling species *Chromis* spp. (planktivore) that clearly prefers exposed sites and the territorial herbivore *S*. *fuscus* that was more abundant in sheltered areas. This pattern is consistent with that of [13], who suggested that swimming ‘ability’ influenced their abundance as *Chromis* has longer bifurcated caudal fins than other pomacentrids. Planktivores are also expected to be more heavily influenced by physical factors related to water motion because zooplankton is often driven by the wind from oceanic to shallow areas [14]. However, wave action may have a strong indirect effect on fish assemblages. The degree of water movement is an important source of variability on other components of the biota [81–85] and also alters the small-scale topographic complexity in exposed relative to sheltered locations, which in turn has direct effects on the composition and relative abundance of species within the fish assemblages [80].

The interaction between boulder size and distance was significant in explaining variation in fish assemblage structure. This interaction means that the slope of one continuous predictor (e.g., boulder size) on the response variable (e.g., fish assemblage) changed as the values of the second continuous predictor (e.g., distance from the river-influenced area) changed [86]. Specifically, this interaction indicates that the greater the distance, the greater the effect of boulder size on fish assemblage. Similarly, the greater the boulder size, the greater the effect of distance on fish assemblage. In this case, it implies that increasing topographic complexity is particularly beneficial for maximizing the positive impact of large distances from riverine influence on fish assemblages. Therefore, this case demonstrates how environmental interactions may pose special challenges in interpreting environmental influences on reef fish assemblages of coastal areas.

The variability of fish assemblage parameters clearly represented a gradient from degraded to healthy reefs. There was a 4.5-fold difference in fish richness and fish trophic group diversity and an 11-fold difference in biomass and 10-fold difference in fish abundance between the distance extremes in our study (1.4 km and 13.1 km; see Fig 5). The lowest fish richness, trophic group diversity and fish abundance were observed in structurally flatter reefs (< bolder size) near islands closer to the rivers (<4.6 km). This finding is consistent with the general observation that abundance, species richness and diversity tend to be a decelerating function of increases in the area of habitat [87]. Second, this pattern suggests that reefs exposed to high sediment loads from local rivers lead to habitat loss due to a decrease in topographic complexity. An increase in sedimentation is associated with a decrease in species richness [15,32,88] through a reduction in the amount of rocky substrata available for settlement of rocky coast organisms [89]. The relationship between biomass and distance from the riverine influence was related to changes in size structure of fish assemblages. Small-sized species, such as the invertebrate feeders *H*. *steindachneri* and *S*. *flaviventris* dominated areas that were closer to the river confluence area, while the opposite trend was observed for larger species such as parrotfishes (e.g., *S*. *frondosum* and *S*. *axillare*) and groupers (e.g., *E*. *marginatus*). The association of larger-sized species with distance is consistent with the expectation that anthropogenic pressure (e.g. effect of river discharges on larger-sized herbivorous fishes) is lower in areas away from land-based activities [29,31]. Although fisheries are important source of changes in biomass [34], our sites are similarly accessible to fishing activities because they are at similar distance from the coast. Therefore, distance from the river confluence is hardly a proxy of fishing pressure in this study.

Major stressors driving reef degradation have included altered trophic structures, whereby multiple specialist groups are replaced by fewer, more generalist groups leading to much simpler ecosystems [8,90–92]. We identified that reefs with the combination of higher riverine influence, lower boulder size and high EAM cover were dominated solely by mobile invertebrate feeders. In contrast, trophic group diversity increased as the distance from the river confluence area increased and as habitats became more structurally complex and with more variable benthic cover (ranging from soft coral to EAM covers). The more diverse substrate increases trophic diversification through an increase in the array of potential food [69], and higher topographic complexity led to greater diversity of algal assemblages through generation of more microenvironments available for algal colonization and growth [93]. Fish-based metrics that are characteristic of a combination of factors and environmental variables may be a valuable tool for managers [94]. The more the habitat characteristics will be recorded precisely, the more accurate the metric will be [95].

We were able to identify assemblages which experience low/infrequent and high/frequent levels of disturbance. In reefs close to rivers, the level of disturbance is too high to permit a more diverse fish assemblage. Species not capable of dealing with increased river discharges (e.g., roving herbivorous and planktivorous) and that are sensitive to the availability and quality of food and shelter resources are not found in such areas. On the other hand, in reefs with low levels of disturbance, competition may be an important ecological force shaping communities. In particular, species with that are inferior competitors for resources may be scarce in less disturbed reefs [96,97]. In these reefs, topographic complexity may have a profound influence on the number of species and permit the coexistence of predator and prey and ontogenetic niche shifts. Our results are consistent with these ideas, as reefs with high topographic complexity associated to large distances from river influences harbor rich and diversified fish assemblages.

### Implications for rocky reef management

This study found that despite the greater variability that existed in topographic complexity and dominant benthic organisms, fish assemblages of insular reefs were more heavily influenced by the distance from the river discharges. It implies that improving water quality is a critical step toward reef restoration. Soil loss from poor land-use practices very often leads to increases in river runoff and suspended solids concentrations that reduce biological diversity on adjacent reefs [31,32]. More than the increase of suspended solids, river runoff may be also a source to the introduction of contaminants into marine protected areas (MPAs). The consequences of these contaminants for fish species can be related to fish reproduction, growth, health and other aspects of life cycle [98,99], and their effects on the fish assemblage structure are still not understood. Our data highlight that investments in MPAs isolated from management initiatives toward coastal conservation could not be effective in long-term. The conservation priorities for reefs exposed to terrestrial inputs should consider, for example, the reduction in sewage or agricultural runoff and finding areas that are suitable for mangrove reforestation in order to improve water quality by restoring the capacity of estuaries to trap sediments [31,100,101]. However, such measures remain rarely implemented in nearshore reef restoration. This is probably because they are labour-intensive, expensive and involve actions that go beyond the jurisdictional boundaries of marine conservation managers.

Management decisions based on key drivers are expected to influence reef recovery (e.g. prioritizing topographically complex reefs, reducing fishing effort or facilitating coral recovery by managing herbivores, see [100,102,103]). However, such decisions may be ineffective in providing local benefits for reefs heavily impacted by rivers, unless integrated watershed management practices are implemented. For instance, the diversity of fish assemblages in Brazilian rocky reefs under anthropogenic disturbances was similar between reefs with very high and very low topographic complexity [29,104]. Moreover, prioritize reefs with higher average boulder size or number of refuges may have limited conservation value if these reefs are subject to strong river discharges. In this sense, great care must be taken to not impair the efficiency of reef conservation measures.

Our results highlight the need for further conservation measures based on the group-specific patterns detected, such as (i) monitoring reefs to understand the factors involved in the dominance of soft coral cover, especially considering that when zoanthids cover large portions of the reef substratum they can actually lower the degree of habitat heterogeneity [105,106] and also to improve the conservation of cryptic assemblages; and (ii) protecting herbivores and carnivores from fishing, considering that structurally complex habitats are related to a greater diversity of trophic functional groups. We also suggest that sites in monitoring programs should be selected only after consideration of the complex relationships among multiple environmental variables and populations instead of using a priori classifications. This practice may help manage reefs at relevant scales of variations. In addition, proposing specific solutions for regions not only defined by geopolitical needs. While many marine conservation decisions are still based on the precautionary principle [107], uncertainties in predicting environmental impacts have been evoqued by economic groups as a reason for the approval environmental licenses [108].

The establishment of MPAs has rarely considered the influence of multiple environmental variables on the distribution of the biota [109,110]. The lack of adequate information in MPA site selection process may be particularly troubling for the conservation of assemblages that are highly variable at small spatial scales (from hundreds of meters to a few kilometers). In this study, islands spaced only 4 km apart likely have drastically different fish assemblages. Therefore, understanding the influence of key environmental predictors on fish assemblages is paramount in MPA planning process, especially when all candidate sites are not feasibly to be assessed. We found that distance from rivers plays an important role in rocky-reef health and thus mitigating the effects of poor land-use practices may be as important as the conservation of reefs itself. Our results indicate that increased distance from river influences, high topographic complexity (namely large boulder size) and EAM cover host a more diversified fish assemblage and maximize the representation of biodiversity. Monitoring biodiversity inexpensively and the inclusion of physical/anthropogenic, biological and topographical variables in a conservation planning are fundamental for the management of spatially-heterogeneous assemblages.

## Supporting Information

S1 Fig River discharge plume on a rocky reef. Reef located 3.3 km from the stating gradient point. Photographs were taken at 01-28-2012 (before rain) and 02-28-2012; after 76 mm rain.
(DOCX)

S1 Table Length–weight relationships parameters used to estimate the fish biomass. (DOCX)

S2 Table Physical, topographical and benthic predictors per site. Mean ±SE of topographical and selected benthic cover predictors (%). Exposure to wave activity: S–sheltered sites; E–exposed sites.
(DOCX)

S3 Table Most discriminating species for fish assemblage structure. Scores for species that best discriminated the first two dbRDA axes and respective trophic and site groups.
(DOCX)

S4 Table List of species by family with trophic group classification. Mean abundance (individuals per 40 m^2^±SE), percent of total observed (%) and frequency of occurrence (FO) of fish species observed in the three site-groups (close, intermediate and far). Groups were defined according to the relationship between fish assemblage structure and environmental predictors.–There is no information available.
(DOCX)

S5 Table Selected species among exposure and site-groups. PERMANOVA results testing the effect of groups (defined according to distribution of the sites along the dbRDA axis), wave exposure and sampling period on the abundances of selected species. df = degrees of freedom, MS = mean sum of squares, F = pseudo-F, *** = *P*<0.001, ** = *P*<0.01, * = *P*<0.05.
(DOCX)

S6 Table Summary of regression analyses between predictors and fish assemblage parameters. **Predictor variables that influenced significantly descriptors of fish assemblage according to simple regression. r, Pearson correlation coefficient. Levels of significance:** ***ear*P*<0.001, ** = *P*<0.01, * = *P*<0.05.
(DOCX)
