# Supplementary figures and images for: The Farther the Better: Effects of Multiple Environmental Variables on Reef Fish Assemblages along a Distance Gradient from River Influences
Source: PLoS One. 2016 Dec 1;11(12):e0166679. doi: 10.1371/journal.pone.0166679 (PMC5131968; doi:10.1371/journal.pone.0166679)

| 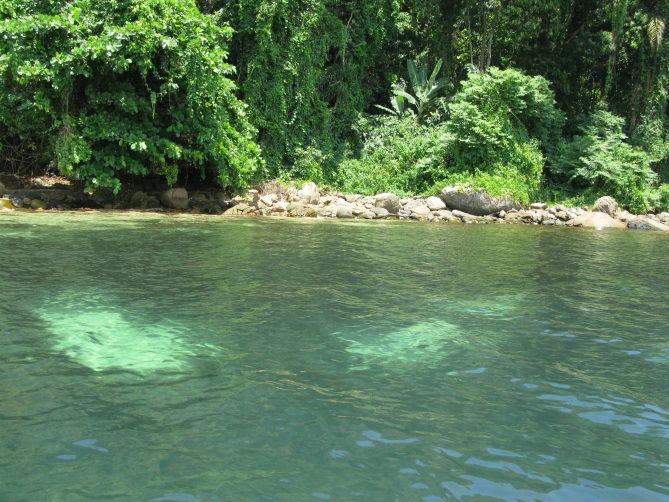 | 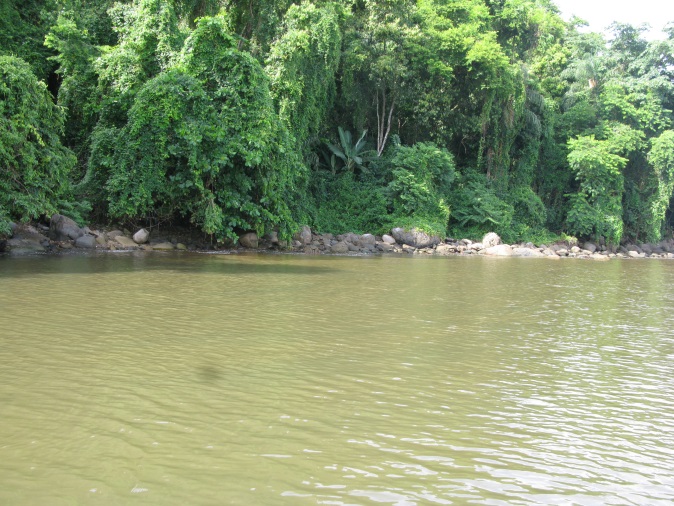 |
| --- | --- |
| Date: 01-28-2012; before rain | Date: 02-28-2012; after 76 mm rain |

**S1 Fig.**

Supplement: S1 Fig — Reef located 3.3 km from the stating gradient point. Photographs were taken at 01-28-2012 (before rain) and 02-28-2012; after 76 mm rain. (DOCX) [file pone.0166679.s001.docx]
