# Supplementary material for: The Farther the Better: Effects of Multiple Environmental Variables on Reef Fish Assemblages along a Distance Gradient from River Influences
Source: PLoS One. 2016 Dec 1;11(12):e0166679. doi: 10.1371/journal.pone.0166679 (PMC5131968; doi:10.1371/journal.pone.0166679)
Supplement: S1 Table — (DOCX) [file pone.0166679.s002.docx]

**S1 Table.**

|  | Coefficients | | Refs | Closest related species/genera |
| --- | --- | --- | --- | --- |
| Family/species | a | b |  |  |
| **Acanthuridae** |  |  |  |  |
| *Acanthurus bahianus* | 0.02370 | 2.975 | [1] |  |
| *Acanthurus chirurgus* | 0.00405 | 3.533 | [1] |  |
| **Atherinopsidae** |  |  |  |  |
| *Atherinella brasiliensis* | 0.00328 | 3.333 | [1] |  |
| **Blenniidae** |  |  |  |  |
| *Parablennius marmoreus* | 0.00430 | 3.52 | [3] | |
| *Scartella cristata* | 0.02810 | 2.414 | [1] |  |
| **Carangidae** |  |  |  |  |
| *Caranx latus* | 0.01860 | 2.856 | [1] |  |
| *Chloroscombrus chrysurus* | 0.01680 | 2.763 | [1] |  |
| *Trachinotus falcatus* | 0.03010 | 2.958 | [1] |  |
| **Chaenopsidae** |  |  |  |  |
| *Emblemariopsis signifer* | 0.00457 | 3.08 | [1] |  |
| **Chaetodontidae** |  |  |  |  |
| *Chaetodon striatus* | 0.02220 | 3.14 | [1] |  |
| **Dactylopteridae** |  |  |  |  |
| *Dactylopterus volitans* | 0.00710 | 3.1 | [1] |  |
| **Diodontidae** |  |  |  |  |
| *Diodon hystrix* | 0.53200 | 2.276 | [1] |  |
| *Chilomycterus spinosus spinosus* | 0.25400 | 2.380 | [1] |  |
| **Elopidae** |  |  |  |  |
| *Elops saurus* | 0.02990 | 2.533 | [1] |  |
| **Epinephelidae** |  |  |  |  |
| *Epinephelus marginatus* | 0.00910 | 3.115 | [1] |  |
| *Mycteroperca acutirostris* | 0.01300 | 3.033 | [1] |  |
| *Mycteroperca microlepis* | 0.02340 | 2.950 | [1] |  |
| *Mycteroperca bonaci* | 0.00684 | 3.205 | [1] |  |
| *Mycteroperca interstitialis* | 0.00090 | 3.582 | [1] |  |
| **Ephippidae** |  |  |  |  |
| *Chaetodipterus faber* | 0.04320 | 2.968 | [1] |  |
| **Gerreidae** |  |  |  |  |
| *Diapterus rhombeus* | 0.00890 | 3.187 | [1] |  |
| *Eucinostomus* spp.* | 0.01060 | 3.03 | [1] | *Eucinostomus argenteus* |
| **Gobiidae** |  |  |  |  |
| *Bathygobius soporator* | 0.01090 | 3.08 | [1] |  |
| *Coryphopterus* spp.* | 0.01310 | 2.967 | [1] | *Coryphopterus glaucofraenum* |
| *Elacatinus figaro* * | 0.00799 | 3.137 | [1] | *Elacatinus oceanops* |
| **Haemulidae** |  |  |  |  |
| *Anisotremus virginicus* | 0.01480 | 3.167 | [1] |  |
| *Anisotremus surinamensis* | 0.02330 | 3.01 | [1] |  |
| *Haemulon steindachneri* | 0.00980 | 3.124 | [1] |  |
|  | Coefficients | | Refs | Closest related species/genera |
| Family/species | a | b |  |  |
| *Haemulon aurolineatum* | 0.01060 | 3.09 | [1] |  |
| *Orthopristis ruber* | 0.00960 | 3.100 | [1] |  |
| **Holocentridae** |  |  |  |  |
| *Holocentrus adscensionis* | 0.02130 | 2.778 | [1] |  |
| **Kyphosidae** |  |  |  |  |
| *Kyphosus* spp.* | 0.01699 | 3.000 | [1] | *Kyphosus incisor* |
| **Labridae** |  |  |  |  |
| *Bodianus pulchellus* * | 0.01440 | 3.053 | [1] | *B. rufus* |
| *Halichoeres poeyi* | 0.00950 | 3.131 | [1] |  |
| *Halichoeres brasiliensis* | 0.00698 | 3.39 | [2] |  |
| *Sparisoma tuiupiranga* * | 0.01210 | 3.028 | [1] | *S. atomarium* |
| *Sparisoma atomarium* | 0.01210 | 3.028 | [1] |  |
| *Cryptotomus roseus* | 0.00780 | 3.275 | [1] |  |
| *Sparisoma frondosum* * | 0.01440 | 3.100 | [1] | *S. axillare* |
| *Sparisoma axillare* | 0.01440 | 3.100 | [1] |  |
| *Sparisoma radians* | 0.01790 | 3.035 | [1] |  |
| *Sparisoma amplum* | 0.01440 | 3.100 | [1] |  |
| *Scarus zelindae* * | 0.01470 | 3.055 | [1] | *Scarus iseri* |
| **Labrisomidae** |  |  |  |  |
| *Labrisomus nuchipinnis* | 0.00840 | 3.200 | [1] |  |
| *Malacoctenus delalandii* | 0.01220 | 2.95 | [2] |  |
| *Paraclinus spectator* | 0.01450 | 2.840 | [1] |  |
| *Starksia brasiliensis* | 0.00710 | 3.15 | [3] |  |
| **Lutjanidae** |  |  |  |  |
| *Lutjanus synagris* | 0.02950 | 2.815 | [1] |  |
| *Ocyurus chrysurus* | 0.08530 | 2.47 | [1] |  |
| **Monacanthidae** |  |  |  |  |
| *Cantherhines macrocerus* | 0.05610 | 2.653 | [1] |  |
| *Cantherhines pullus* | 0.06840 | 2.563 | [1] |  |
| *Stephanolepis hispidus* | 0.03329 | 2.772 | [1] |  |
| **Mugilidae** |  |  |  |  |
| *Mugil curema* | 0.01080 | 2.969 | [1] |  |
| **Mullidae** |  |  |  |  |
| *Pseudupeneus maculatus* | 0.01500 | 3.157 | [1] |  |
| **Muraenidae** |  |  |  |  |
| *Gymnothorax ocellatus* | 0.00027 | 3.446 | [1] |  |
| **Myliobatidae** |  |  |  |  |
| *Aetobatus narinari* | 0.00586 | 3.13 | [1] |  |
| **Ostraciidae** |  |  |  |  |
| *Acanthostracion polygonius* | 0.10500 | 2.690 | [1] |  |
| **Pempheridae** |  |  |  |  |
| *Pempheris schomburgkii* | 0.04390 | 2.620 | [1] |  |
| **Pomacanthidae** |  |  |  |  |
| *Pomacanthus paru* | 0.02030 | 3.126 | [1] |  |
|  | Coefficients | | Refs | Closest related species/genera |
| Family/species | a | b |  |  |
| **Pomacentridae** |  |  |  |  |
| *Abudefduf saxatilis* | 0.02270 | 3.142 | [1] |  |
| *Chromis multilineata* * | 0.02990 | 2.793 | [1] | *Chromis chromis* |
| *Stegastes fuscus* | 0.01930 | 2.960 | [1] |  |
| *Stegastes pictus* * | 0.01930 | 2.96 | [1] | *S. fuscus* |
| *Stegastes variabilis* | 0.03240 | 2.836 | [1] |  |
| **Priacanthidae** |  |  |  |  |
| *Heteropriacanthus cruentatus* | 0.02793 | 2823 | [1] |  |
| **Sciaenidae** |  |  |  |  |
| *Odontoscion dentex* | 0.01050 | 3.007 | [1] |  |
| *Pareques acuminatus* | 0.00870 | 3.202 | [1] |  |
| **Scorpaenidae** |  |  |  |  |
| *Scorpaena plumieri* | 0.02430 | 2.949 | [1] |  |
| **Serranidae** |  |  |  |  |
| *Serranus flaviventris* | 0.00790 | 3.450 | [1] |  |
| *Serranus baldwini* * | 0.00790 | 3.450 | [1] | *Serranus flaviventris* |
| **Sparidae** |  |  |  |  |
| *Archosargus rhomboidalis* | 0.02150 | 2.960 | [1] |  |
| *Calamus penna* | 0.09770 | 2.542 | [1] |  |
| *Diplodus argenteus* * | 0.01382 | 3.070 | [1] | *Diplodus sargus sargus* |
| **Tetraodontidae** |  |  |  |  |
| *Canthigaster figueiredoi* * | 0.02989 | 2.979 | [1] | *Canthigaster solandri* |
| *Sphoeroides spengleri* | 0.04200 | 2.610 | [1] |  |
| *Sphoeroides testudineus* | 0.02290 | 3.008 | [1] |  |
| *Sphoeroides greeleyi* | 0.01770 | 3.086 | [1] |  |

* Species with no coefficients available in the literature. When coefficient values were not recorded for a species, we used coefficients for the closest related species or genera.

[1] Froese R, Pauly D. Fishbase. World Wide Web electronic publication; 2014. Accessed: <http://www.fishbase.org>.

[2] Macieira RM, Joyeux JC. Length–weight relationships for rockpool ﬁshes in Brazil. J Appl Ichthyol. 2008;25(3): 358–359. doi: 10.1111/j.1439-0426.2008.01118.x.

[3] Camilato V, Simon T, Pinheiro HT, Pimentel CR, Joyeux JC. Length-weight relationships for some cryptobenthic reef ﬁshes oﬀ Guarapari. J Appl Ichthyol. 2010;26(3): 463–464. doi: 10.1111/j.1439-0426.2009.01343.x.
