# Supplementary material for: The Farther the Better: Effects of Multiple Environmental Variables on Reef Fish Assemblages along a Distance Gradient from River Influences
Source: PLoS One. 2016 Dec 1;11(12):e0166679. doi: 10.1371/journal.pone.0166679 (PMC5131968; doi:10.1371/journal.pone.0166679)
Supplement: S2 Table — Mean ±SE of topographical and selected benthic cover predictors (%). Exposure to wave activity: S–sheltered sites; E–exposed sites. (DOCX) [file pone.0166679.s003.docx]

**S2 Table.**

| Distance (Km) | Number of samples | | Exposure | Refuges | Boulder size | Turf | Fleshy algae | Soft coral |
| --- | --- | --- | --- | --- | --- | --- | --- | --- |
|  | Summer of 2011 | Summer of 2012 |  |  |  |  |  |  |
| 1.4 | 6 | 9 | S | 1.5±0.1 | 34.5±0.9 | 95.2±1.1 | 2.5±0.6 | - |
| 1.8 | 6 | 9 | E | 1.6±0.2 | 35.5±1.6 | 72.7±2.5 | 24.6±2.4 | - |
| 3.3 | 12 | 12 | S | 1.9±0.2 | 41.6±1.2 | 84.6±1.7 | 4.6±0.8 | 2.9±1.5 |
| 3.4 | 12 | 9 | E | 1.6±0.2 | 29.8±0.6 | 83.9±1.5 | 10.7±1.2 | - |
| 3.7 | 6 | 9 | S | 0.5±0.2 | 77.9±5.2 | 68.1±2.6 | 25.2±2.4 | 1.1±0.7 |
| 3.9 | 6 | 12 | E | 1.1±0.1 | 36.8±1.7 | 37.9±2.1 | 48.8±2.3 | 10.2±2.3 |
| 4.4 | 12 | 15 | S | 3.2±0.2 | 53.1±1.4 | 60.1±2.0 | 28.2±2.2 | 7.7±1.3 |
| 4.6 | 6 | 9 | E | 0.7±0.2 | 80.8±5.4 | 68.7±2.9 | 17.9±2.3 | 3.5±1.8 |
| 8.1 | 6 | 6 | S | 3.6±0.3 | 44.9±2.6 | 18.0±2.4 | 3.1±0.7 | 69.7±3.4 |
| 8.7 | 6 | 6 | E | 2.7±0.3 | 83.9±9.6 | 30.7±3.4 | 20.3±3.9 | 43.7±4.2 |
| 9 | 6 | 3 | S | 4.1±0.3 | 48.9±2.6 | 22.4±3.1 | 7.8±1.9 | 67.1±4.1 |
| 9.2 | 6 | 9 | E | 3.3±0.3 | 69.7±3.6 | 26.1±4.4 | 4.0±1.4 | 65.9±4.7 |
| 11.2 | 6 | 9 | S | 1.4±0.2 | 58.9±2.8 | 52.3±3.7 | 33.9±3.5 | 12.1±3.3 |
| 11.5 | 6 | 12 | E | 0.8±0.1 | 106.7±11.6 | 36.1±3.3 | 36.9±3.8 | 22.0±4.2 |
| 12.8 | 6 | 3 | S | 3.5±0.3 | 56.9±2.7 | 31.2±2.8 | 4.9±0.9 | 54.5±3.2 |
| 13.1 | 6 | 6 | E | 0.4±0.1 | 119±1.3 | 79.7±3.2 | 7.4±1.4 | 7.9±3.1 |
| Total | 114 | 138 |  |  |  |  |  |  |
