# Supplementary material for: The Farther the Better: Effects of Multiple Environmental Variables on Reef Fish Assemblages along a Distance Gradient from River Influences
Source: PLoS One. 2016 Dec 1;11(12):e0166679. doi: 10.1371/journal.pone.0166679 (PMC5131968; doi:10.1371/journal.pone.0166679)
Supplement: S3 Table — Scores for species that best discriminated the first two dbRDA axes and respective trophic and site groups. (DOCX) [file pone.0166679.s004.docx]

**S3 Table.**

| Species | dbRDA1 | dbRDA2 | Trophic group | Site-group |
| --- | --- | --- | --- | --- |
| *Haemulon steindachneri* | 0.86 |  | Mobile invertebrate feeder | Close |
| *Serranus flaviventris* | 0.82 |  | Mobile invertebrate feeder | Close |
| *Sphoeroides greeleyi* | 0.7 |  | Mobile invertebrate feeder | Close |
| *Sphoeroides testudineus* | 0.45 |  | Omnivorous | Close |
| *Eucinostomus* sp. | 0.41 |  | Mobile invertebrate feeder | Close |
| *Dactylopterus volitans* | 0.4 |  | Mobile invertebrate feeder | Close |
| *Orthopristis ruber* | 0.39 |  | Mobile invertebrate feeder | Close |
| *Stephanolepis hispidus* | 0.39 |  | Omnivorous | Close |
| *Diapterus rhombeus* | 0.31 |  | Mobile invertebrate feeder | Close |
| *Lutjanus synagris* | 0.31 |  | Carnivore | Close |
| *Bathygobius soporator* | 0.3 |  | Mobile invertebrate feeder | Close |
| *Stegastes fuscus* | -0.62 | 0.6 | Territorial herbivore | Intermediate/Far |
| *Coryphopterus spp.* |  | 0.48 | Mobile invertebrate feeder | Intermediate |
| *Emblemariopsis signifer* |  | 0.39 | Mobile invertebrate feeder | Intermediate |
| *Scartella cristata* |  | 0.35 | Territorial herbivore | Intermediate |
| *Chaetodon striatus* | -0.82 |  | Sessile invertebrate feeder | Far |
| *Abudefduf saxatilis* | -0.78 |  | Omnivorous | Far |
| *Halichoeres poeyi* | -0.77 | -0.41 | Mobile invertebrate feeder | Far |
| *Pomacanthus paru* | -0.76 |  | Omnivorous | Far |
| *Holocentrus adscensionis* | -0.75 |  | Mobile invertebrate feeder | Far |
| *Sparisoma frondosum* | -0.7 | -0.48 | Roving herbivore | Far |
| *Haemulon aurolineatum* | -0.7 |  | Mobile invertebrate feeder | Far |
| *Epinephelus marginatus* | -0.65 |  | Carnivore | Far |
| *Parablennius marmoreus* | -0.65 |  | Omnivorous | Far |
| *Acanthurus chirurgus* | -0.62 | -0.32 | Roving herbivore | Far |
| *Chromis mulitilineata* | -0.58 | -0.42 | Planktivores | Far |
| *Canthigaster figueiredoi* | -0.54 | -0.37 | Sessile invertebrate feeder | Far |
| *Kyphosus* spp. | -0.52 | -0.47 | Roving herbivore | Far |
| *Halichoeres brasiliensis* | -0.51 | -0.44 | Mobile invertebrate feeder | Far |
| *Pareques acuminatus* | -0.51 | -0.57 | Mobile invertebrate feeder | Far |
| *Sparisoma axillare* | -0.51 | -0.52 | Roving herbivore | Far |
| *Labrisomus nuchipinnis* | -0.48 | -0.59 | Mobile invertebrate feeder | Far |
| *Anisotremus virginicus* | -0.46 |  | Mobile invertebrate feeder | Far |
| *Sparisoma amplum* | -0.45 | -0.35 | Roving herbivore | Far |
| *Diplodus argenteus* | -0.44 | -0.42 | Omnivorous | Far |
| *Acanthurus bahianus* | -0.42 | -0.38 | Roving herbivore | Far |
| *Scarus zelindae* | -0.41 | -0.46 | Roving herbivore | Far |
| *Chaetodipterus faber* | -0.4 | -0.36 | Omnivorous | Far |
| *Odontoscion dentex* | -0.39 |  | Carnivore | Far |
| *Pseudupeneus maculatus* | -0.39 |  | Mobile invertebrate feeder | Far |
| *Mycteroperca acutirostris* | -0.36 |  | Carnivore | Far |
| *Sparisoma tuiupiranga* | -0.36 | -0.37 | Roving herbivore | Far |
| *Pempheris schomburgkii* | -0.34 |  | Planktivores | Far |
| *Cantherhines pullus* | -0.33 | -0.44 | Omnivore | Far |
| *Chilomycterus spinosus spinosus* | -0.33 | -0.35 | Mobile invertebrate feeder | Far |
| *Elacatinus figaro* | -0.33 |  | Mobile invertebrate feeder | Far |
| *Stegastes variabilis* | -0.32 | -0.57 | Territorial herbivore | Far |
| *Stegastes pictus* | -0.32 |  | Territorial herbivore | Far |
| *Malacoctenus delalandii* |  | -0.49 | Mobile invertebrate feeder | Far |
| *Diodon hystrix* |  | -0.39 | Mobile invertebrate feeder | Far |
| *Bodianus pulchellus* |  | -0.39 | Mobile invertebrate feeder | Far |
