# Supplementary material for: The Farther the Better: Effects of Multiple Environmental Variables on Reef Fish Assemblages along a Distance Gradient from River Influences
Source: PLoS One. 2016 Dec 1;11(12):e0166679. doi: 10.1371/journal.pone.0166679 (PMC5131968; doi:10.1371/journal.pone.0166679)
Supplement: S4 Table — Mean abundance (individuals per 40 m2±SE), percent of total observed (%) and frequency of occurrence (FO) of fish species observed in the three site-groups (close, intermediate and far). Groups were defined according to the relationship between fish assemblage structure and environmental predictors.–There is no information available. (DOCX) [file pone.0166679.s005.docx]

**S4 Table.**

| Family/Species | Trophic group |  | Close (< 5 Km) | | |  | Intermediate (5 – 10 Km) | | |  | Far (10 – 13 Km) | | |
| --- | --- | --- | --- | --- | --- | --- | --- | --- | --- | --- | --- | --- | --- |
|  |  |  | Abundance | % | FO |  | Abundance | % | FO |  | Abundance | % | FO |
| **Acanthuridae** |  |  |  |  |  |  |  |  |  |  |  |  |  |
| *Acanthurus bahianus* | Rov. Herbiv. |  |  |  |  |  | 0.03±0.03 | 0.06 | 2.09 |  | 0.08±0.05 | 0.09 | 5.56 |
| *Acanthurus chirurgus* | Rov. Herbiv. |  | 0.05±0.03 | 0.11 | 3.34 |  | 0.34±0.2 | 0.86 | 12.5 |  | 0.73±0.26 | 0.87 | 31.49 |
| **Atherinopsidae** |  |  |  |  |  |  |  |  |  |  |  |  |  |
| *Atherinella brasiliensis* | Omnivore |  | 0.12±0.12 | 0.28 | 0.67 |  |  |  |  |  |  |  |  |
| **Blenniidae** |  |  |  |  |  |  |  |  |  |  |  |  |  |
| *Parablennius marmoreus* | Omnivore |  | 0.01±0.01 | 0.02 | 0.67 |  | 0.17±0.08 | 0.43 | 12.5 |  | 0.23±0.08 | 0.27 | 16.67 |
| *Scartella cristata* | Ter. Herbiv. |  |  |  |  |  | 0.11±0.06 | 0.27 | 8.34 |  | 0.06±0.05 | 0.07 | 3.71 |
| **Carangidae** |  |  |  |  |  |  |  |  |  |  |  |  |  |
| *Caranx latus* | Carnivore |  |  |  |  |  |  |  |  |  | 0.02±0.02 | 0.03 | 1.86 |
| *Chloroscombrus chrysurus* | Carnivore |  | 0.02±0.01 | 0.04 | 1.34 |  |  |  |  |  |  |  |  |
| *Trachinotus falcatus* | Carnivore |  |  |  |  |  |  |  |  |  | 0.02±0.02 | 0.03 | 1.86 |
| **Chaenopsidae** |  |  |  |  |  |  |  |  |  |  |  |  |  |
| *Emblemariopsis signifer* | Mob. Invert. |  | 0.06±0.03 | 0.13 | 3.34 |  | 0.36±0.11 | 0.91 | 20.84 |  |  |  |  |
| **Chaetodontidae** |  |  |  |  |  |  |  |  |  |  |  |  |  |
| *Chaetodon striatus* | Sessile Invert. |  | 0.27±0.05 | 0.61 | 19.34 |  | 0.88±0.16 | 2.24 | 52.09 |  | 1.15±0.14 | 1.38 | 64.82 |
| **Dactylopteridae** |  |  |  |  |  |  |  |  |  |  |  |  |  |
| *Dactylopterus volitans* | Mob. Invert. |  | 0.08±0.03 | 0.17 | 6.67 |  |  |  |  |  |  |  |  |
| **Diodontidae** |  |  |  |  |  |  |  |  |  |  |  |  |  |
| *Diodon hystrix* | Mob. Invert. |  |  |  |  |  |  |  |  |  | 0.02±0.02 | 0.03 | 1.86 |
| *Chilomycterus spinosus spinosus* | Mob. Invert. |  | 0.02±0.02 | 0.05 | 2 |  |  |  |  |  | 0.12±0.06 | 0.14 | 9.26 |
| **Elopidae** |  |  |  |  |  |  |  |  |  |  |  |  |  |
| *Elops saurus* | Carnivore |  | 0.01±0.01 | 0.02 | 0.67 |  |  |  |  |  |  |  |  |
| Family/Species | Trophic group |  | Close (< 5 Km) | | |  | Intermediate (5 – 10 Km) | | |  | Far (10 – 13 Km) | | |
|  |  |  | Abundance | % | FO |  | Abundance | % | FO |  | Abundance | % | FO |
| **Epinephelidae** |  |  |  |  |  |  |  |  |  |  |  |  |  |
| *Epinephelus marginatus* | Carnivore |  | 0.01±0.01 | 0.02 | 0.67 |  | 0.25±0.09 | 0.64 | 18.75 |  | 0.32±0.09 | 0.38 | 22.23 |
| *Mycteroperca acutirostris* | Carnivore |  | 0.36±0.06 | 0.8 | 28.67 |  | 0.53±0.12 | 1.34 | 35.42 |  | 0.56±0.1 | 0.67 | 44.45 |
| *Mycteroperca microlepis* | Carnivore |  | 0.02±0.01 | 0.04 | 1.34 |  |  |  |  |  |  |  |  |
| *Mycteroperca bonaci* | Carnivore |  | 0.01±0.01 | 0.02 | 0.67 |  | 0.05±0.03 | 0.11 | 4.17 |  | 0.04±0.03 | 0.05 | 3.71 |
| *Mycteroperca interstitialis* | Carnivore |  | 0.01±0.01 | 0.02 | 0.67 |  |  |  |  |  |  |  |  |
| **Ephippidae** |  |  |  |  |  |  |  |  |  |  |  |  |  |
| *Chaetodipterus faber* | Omnivore |  | 0.01±0.01 | 0.02 | 0.67 |  | 0.05±0.05 | 0.11 | 2.09 |  | 0.15±0.1 | 0.18 | 7.41 |
| **Gerreidae** |  |  |  |  |  |  |  |  |  |  |  |  |  |
| *Diapterus rhombeus* | Mob. Invert. |  | 0.11±0.09 | 0.25 | 2.67 |  | 0.03±0.03 | 0.06 | 2.09 |  |  |  |  |
| *Eucinostomus* spp. | Mob. Invert. |  | 4.62±1.56 | 10.41 | 19.34 |  |  |  |  |  |  |  |  |
| **Gobiidae** |  |  |  |  |  |  |  |  |  |  |  |  |  |
| *Bathygobius soporator* | Mob. Invert. |  | 0.14±0.07 | 0.32 | 4 |  |  |  |  |  |  |  |  |
| *Coryphopterus* spp. | Mob. Invert. |  | 0.56±0.12 | 1.27 | 20.67 |  | 1.17±0.5 | 2.99 | 31.25 |  | 0.06±0.05 | 0.07 | 3.71 |
| *Elacatinus figaro* | Mob. Invert. |  | 0.1±0.04 | 0.22 | 5.34 |  | 0.34±0.11 | 0.86 | 22.92 |  | 1.86±0.96 | 2.23 | 22.23 |
| **Haemulidae** |  |  |  |  |  |  |  |  |  |  |  |  |  |
| *Anisotremus virginicus* | Mob. Invert. |  | 0.3±0.06 | 0.67 | 18 |  | 0.53±0.13 | 1.34 | 35.42 |  | 0.65±0.11 | 0.78 | 50 |
| *Anisotremus surinamensis* | Mob. Invert. |  | 0.16±0.04 | 0.35 | 11.34 |  | 0.3±0.12 | 0.75 | 16.67 |  | 0.12±0.06 | 0.14 | 9.26 |
| *Haemulon steindachneri* | Mob. Invert./Plankt. |  | 19.3±1.81 | 43.52 | 92 |  | 3.42±0.84 | 8.74 | 62.5 |  | 0.02±0.02 | 0.03 | 1.86 |
| *Haemulon aurolineatum* | Mob. Invert./ Plankt. |  | 7.28±1.51 | 16.4 | 45.34 |  | 3.88±0.59 | 9.91 | 85.42 |  | 23.36±5.86 | 28.05 | 87.04 |
| *Orthopristis ruber* | Mob. Invert. |  | 0.27±0.06 | 0.61 | 17.34 |  | 0.17±0.07 | 0.43 | 12.5 |  | 0.06±0.06 | 0.07 | 1.86 |
| **Holocentridae** |  |  |  |  |  |  |  |  |  |  |  |  |  |
| *Holocentrus adscensionis* | Mob. Invert. |  | 0.01±0.01 | 0.02 | 0.67 |  | 0.3±0.12 | 0.75 | 18.75 |  | 0.8±0.16 | 0.96 | 51.86 |
| **Kyphosidae** |  |  |  |  |  |  |  |  |  |  |  |  |  |
| *Kyphosus* spp. | Rov. Herbiv. |  |  |  |  |  |  |  |  |  | 0.39±0.29 | 0.47 | 9.26 |
| **Labridae** |  |  |  |  |  |  |  |  |  |  |  |  |  |
| Family/Species | Trophic group |  | Close (< 5 Km) | | |  | Intermediate (5 – 10 Km) | | |  | Far (10 – 13 Km) | | |
|  |  |  | Abundance | % | FO |  | Abundance | % | FO |  | Abundance | % | FO |
| *Bodianus pulchellus* | Mob. Invert. |  |  |  |  |  |  |  |  |  | 0.02±0.02 | 0.03 | 1.86 |
| *Halichoeres poeyi* | Mob. Invert. |  | 0.01±0.01 | 0.02 | 0.67 |  | 0.15±0.06 | 0.38 | 12.5 |  | 1.67±0.21 | 2.01 | 77.78 |
| *Halichoeres brasiliensis* | Mob. Invert. |  |  |  |  |  |  |  |  |  | 0.15±0.08 | 0.18 | 9.26 |
| *Sparisoma tuiupiranga* | Rov. Herbiv. |  |  |  |  |  | 0.13±0.13 | 0.32 | 2.09 |  | 0.23±0.14 | 0.27 | 9.26 |
| *Sparisoma atomarium* | Rov. Herbiv. |  |  |  |  |  |  |  |  |  | 0.02±0.02 | 0.03 | 1.86 |
| *Cryptotomus roseus* | Rov. Herbiv. |  |  |  |  |  |  |  |  |  | 0.12±0.06 | 0.14 | 9.26 |
| *Sparisoma frondosum* | Rov. Herbiv. |  |  |  |  |  | 0.15±0.06 | 0.38 | 12.5 |  | 1.95±0.44 | 2.34 | 59.26 |
| *Sparisoma axillare* | Rov. Herbiv. |  | 0.03±0.02 | 0.07 | 1.34 |  |  |  |  |  | 0.41±0.14 | 0.49 | 22.23 |
| *Sparisoma radians* | Rov. Herbiv. |  |  |  |  |  | 0.03±0.03 | 0.06 | 2.09 |  | 0.25±0.08 | 0.29 | 18.52 |
| *Sparisoma amplum* | Rov. Herbiv. |  |  |  |  |  |  |  |  |  | 0.06±0.04 | 0.07 | 5.56 |
| *Scarus zelindae* | Rov. Herbiv. |  |  |  |  |  |  |  |  |  | 0.15±0.07 | 0.18 | 11.12 |
| **Labrisomidae** |  |  |  |  |  |  |  |  |  |  |  |  |  |
| *Labrisomus nuchipinnis* | Mob. Invert. |  | 0.02±0.01 | 0.04 | 1.34 |  | 0.03±0.03 | 0.06 | 2.09 |  | 0.23±0.07 | 0.27 | 18.52 |
| *Malacoctenus delalandii* | Mob. Invert. |  | 0.61±0.11 | 1.37 | 32 |  | 0.21±0.09 | 0.54 | 14.59 |  | 0.86±0.17 | 1.03 | 48.15 |
| *Paraclinus spectator* | Mob. Invert. |  | 0.01±0.01 | 0.02 | 0.67 |  |  |  |  |  |  |  |  |
| *Starksia brasiliensis* | – |  |  |  |  |  |  |  |  |  | 0.02±0.02 | 0.03 | 1.86 |
| **Lutjanidae** |  |  |  |  |  |  |  |  |  |  |  |  |  |
| *Lutjanus synagris* | Carnivore |  | 0.04±0.02 | 0.08 | 3.34 |  |  |  |  |  |  |  |  |
| *Ocyurus chrysurus* | Carnivore |  |  |  |  |  |  |  |  |  | 0.02±0.02 | 0.03 | 1.86 |
| **Monacanthidae** |  |  |  |  |  |  |  |  |  |  |  |  |  |
| *Cantherhines macrocerus* | Omnivore |  |  |  |  |  | 0.05±0.03 | 0.11 | 4.17 |  |  |  |  |
| *Cantherhines pullus* | Omnivore |  |  |  |  |  |  |  |  |  | 0.06±0.04 | 0.07 | 5.56 |
| *Stephanolepis hispidus* | Omnivore |  | 0.18±0.04 | 0.41 | 16 |  | 0.11±0.06 | 0.27 | 8.34 |  | 0.02±0.02 | 0.03 | 1.86 |
| **Mugilidae** |  |  |  |  |  |  |  |  |  |  |  |  |  |
| *Mugil curema* | Plankt. |  | 0.4±0.21 | 0.91 | 3.34 |  | 0.63±0.63 | 1.6 | 2.09 |  |  |  |  |
| **Mullidae** |  |  |  |  |  |  |  |  |  |  |  |  |  |
| Family/Species | Trophic group |  | Close (< 5 Km) | | |  | Intermediate (5 – 10 Km) | | |  | Far (10 – 13 Km) | | |
|  |  |  | Abundance | % | FO |  | Abundance | % | FO |  | Abundance | % | FO |
| *Pseudupeneus maculatus* | Mob. Invert. |  | 0.01±0.01 | 0.02 | 0.67 |  | 0.07±0.04 | 0.16 | 6.25 |  | 0.08±0.04 | 0.09 | 7.41 |
| **Muraenidae** |  |  |  |  |  |  |  |  |  |  |  |  |  |
| *Gymnothorax ocellatus* | Carnivore |  |  |  |  |  |  |  |  |  | 0.04±0.03 | 0.05 | 3.71 |
| **Myliobatidae** |  |  |  |  |  |  |  |  |  |  |  |  |  |
| *Aetobatus narinari* | Carnivore |  |  |  |  |  |  |  |  |  | 0.02±0.02 | 0.03 | 1.86 |
| **Ostraciidae** |  |  |  |  |  |  |  |  |  |  |  |  |  |
| *Acanthostracion polygonius* | Omnivore |  |  |  |  |  | 0.05±0.03 | 0.11 | 4.17 |  | 0.02±0.02 | 0.03 | 1.86 |
| **Pempheridae** |  |  |  |  |  |  |  |  |  |  |  |  |  |
| *Pempheris schomburgkii* | Plankt. |  | 0.01±0.01 | 0.02 | 0.67 |  | 0.07±0.04 | 0.16 | 6.25 |  | 4±2.22 | 4.81 | 14.82 |
| **Pomacanthidae** |  |  |  |  |  |  |  |  |  |  |  |  |  |
| *Pomacanthus paru* | Omnivore |  | 0.01±0.01 | 0.02 | 0.67 |  | 0.19±0.07 | 0.48 | 16.67 |  | 0.58±0.11 | 0.69 | 40.75 |
| **Pomacentridae** |  |  |  |  |  |  |  |  |  |  |  |  |  |
| *Abudefduf saxatilis* | Omnivore |  | 5.07±0.81 | 11.43 | 66.67 |  | 19.23±2.48 | 49.18 | 100 |  | 35.69±5.33 | 42.86 | 94.45 |
| *Chromis multilineata* | Plankt. |  |  |  |  |  | 0.05±0.03 | 0.11 | 4.17 |  | 0.21±0.09 | 0.25 | 12.97 |
| *Stegastes fuscus* | Ter. Herbiv. |  | 0.34±0.07 | 0.77 | 22.67 |  | 3.07±0.34 | 7.84 | 89.59 |  | 1.49±0.23 | 1.78 | 70.38 |
| *Stegastes pictus* | Ter. Herbiv. |  |  |  |  |  | 0.03±0.03 | 0.06 | 2.09 |  | 0.02±0.02 | 0.03 | 1.86 |
| *Stegastes variabilis* | Ter. Herbiv. |  | 0.04±0.02 | 0.1 | 4 |  |  |  |  |  | 0.17±0.07 | 0.21 | 12.97 |
| **Priacanthidae** |  |  |  |  |  |  |  |  |  |  |  |  |  |
| *Heteropriacanthus cruentatus* | Mob. Invert. |  | 0.02±0.02 | 0.05 | 1.34 |  | 1.05±1.05 | 2.67 | 2.09 |  |  |  |  |
| **Sciaenidae** |  |  |  |  |  |  |  |  |  |  |  |  |  |
| *Odontoscion dentex* | Carnivore |  | 0.02±0.01 | 0.04 | 1.34 |  | 0.07±0.04 | 0.16 | 6.25 |  | 0.6±0.44 | 0.72 | 9.26 |
| *Pareques acuminatus* | Mob. Invert. |  | 0.05±0.02 | 0.11 | 4 |  | 0.03±0.03 | 0.06 | 2.09 |  | 0.34±0.08 | 0.41 | 27.78 |
| **Scorpaenidae** |  |  |  |  |  |  |  |  |  |  |  |  |  |
| *Scorpaena plumieri* | Carnivore |  | 0.03±0.02 | 0.07 | 2.67 |  | 0.11±0.05 | 0.27 | 10.42 |  | 0.04±0.03 | 0.05 | 3.71 |
| **Serranidae** |  |  |  |  |  |  |  |  |  |  |  |  |  |
| *Serranus flaviventris* | Mob. Invert. |  | 1.71±0.22 | 3.85 | 60.67 |  | 0.17±0.07 | 0.43 | 12.5 |  |  |  |  |
| Family/Species | Trophic group |  | Close (< 5 Km) | | |  | Intermediate (5 – 10 Km) | | |  | Far (10 – 13 Km) | | |
|  |  |  | Abundance | % | FO |  | Abundance | % | FO |  | Abundance | % | FO |
| *Serranus baldwini* | Mob. Invert. |  |  |  |  |  |  |  |  |  | 0.12±0.07 | 0.14 | 7.41 |
| **Sparidae** |  |  |  |  |  |  |  |  |  |  |  |  |  |
| *Archosargus rhomboidalis* | Omnivore |  | 0.06±0.04 | 0.13 | 2.67 |  |  |  |  |  |  |  |  |
| *Calamus penna* | Omnivore |  |  |  |  |  |  |  |  |  | 0.02±0.02 | 0.03 | 1.86 |
| *Diplodus argenteus* | Omnivore |  | 1.07±0.32 | 2.41 | 23.34 |  | 0.55±0.19 | 1.39 | 25 |  | 2.73±0.92 | 3.27 | 51.86 |
| **Tetraodontidae** |  |  |  |  |  |  |  |  |  |  |  |  |  |
| *Canthigaster figueiredoi* | Sessile invert. |  |  |  |  |  | 0.03±0.03 | 0.06 | 2.09 |  | 0.1±0.04 | 0.12 | 9.26 |
| *Sphoeroides spengleri* | Mob. Invert. |  | 0.08±0.03 | 0.17 | 6 |  | 0.13±0.05 | 0.32 | 12.5 |  | 0.06±0.05 | 0.07 | 3.71 |
| *Sphoeroides testudineus* | Omnivore |  | 0.1±0.04 | 0.22 | 7.34 |  |  |  |  |  | 0.02±0.02 | 0.03 | 1.86 |
| *Sphoeroides greeleyi* | Mob. Invert. |  | 0.7±0.1 | 1.58 | 40 |  | 0.05±0.03 | 0.11 | 4.17 |  |  |  |  |
