# Supplementary material for: The Farther the Better: Effects of Multiple Environmental Variables on Reef Fish Assemblages along a Distance Gradient from River Influences
Source: PLoS One. 2016 Dec 1;11(12):e0166679. doi: 10.1371/journal.pone.0166679 (PMC5131968; doi:10.1371/journal.pone.0166679)
Supplement: S5 Table — PERMANOVA results testing the effect of groups (defined according to distribution of the sites along the dbRDA axis), wave exposure and sampling period on the abundances of selected species. df = degrees of freedom, MS = mean sum of squares, F = pseudo-F, *** = P<0.001, ** = P<0.01, * = P<0.05. (DOCX) [file pone.0166679.s006.docx]

**S5 Table.**

| Species |  | *Abudefduf*  *saxatilis* | | | | |  | *Holocentrus adscensionis* | | | | |  | *Haemulon steindachneri* | | | | |  | *Haemulon aurolineatum* | | | |
| --- | --- | --- | --- | --- | --- | --- | --- | --- | --- | --- | --- | --- | --- | --- | --- | --- | --- | --- | --- | --- | --- | --- | --- |
| Source | *df* | MS | | F | | *P* |  | MS | F | | *P* | |  | MS | | F | | *P* |  | MS | F | *P* | |
| Groups | 2 | 2154 | | 54.9 | | *** |  | 11.2 | 30.8 | | *** | |  | 9070.4 | | 33.3 | | *** |  | 6085.2 | 10.2 | *** | |
| Exposure | 1 | 6451 | | 16.4 | | *** |  | 5.4 | 14.8 | | *** | |  | 790.9 | | 2.9 | | ns |  | 370.7 | 0.6 | ns | |
| Time | 1 | 2200.5 | | 5.6 | | * |  | 0.01 | <0.1 | | ns | |  | 1396.5 | | 5.1 | | * |  | 1516.9 | 2.5 | ns | |
| Gr*Ex | 2 | 1755.6 | | 4.4 | | * |  | 4.5 | 12.3 | | *** | |  | 482.3 | | 1.8 | | ns |  | 132.7 | 0.2 | ns | |
| Gr*Ti | 2 | 233.9 | | 0.6 | | ns |  | 2.3 | <0.1 | | *** | |  | 593.2 | | 2.2 | | ns |  | 1738 | 2.9 | ns | |
| Ex*Ti | 1 | 1155.1 | | 2.9 | | ns |  | <0.1 | <0.1 | | ns | |  | 53.3 | | 0.2 | | ns |  | 1201.7 | 2.0 | ns | |
| Gr*Ex*Ti | 2 | 1291.6 | | 3.3 | | * |  | <0.1 | <0.1 | | ns | |  | 14.7 | | 0.1 | | ns |  | 626.6 | 1.0 | ns | |
| Residuals | 240 | 392.3 | |  | |  |  | 0.4 |  | |  | |  | 272.2 | |  | |  |  | 595.7 |  |  | |
| Total | 251 |  | |  | |  |  |  |  | |  | |  |  | |  | |  |  |  |  |  | |
|  |  |  | |  | |  |  |  |  | |  | |  |  | |  | |  |  |  |  |  | |
| Species |  | *Serranus flaviventris* | | | | |  | *Chaetodon striatus* | | | | |  | *Sphoeroides greeleyi* | | | | |  | *Halichoeres poeyi* | | | |
| Source | *df* | MS | | F | | *P* |  | MS | F | | *P* | |  | MS | F | | *P* | |  | MS | F | | *P* |
| Groups | 2 | 79.1 | | 17.9 | | *** |  | 16.5 | 26.6 | | *** | |  | 13.5 | 17.5 | | *** | |  | 52.3 | 112.1 | | *** |
| Exposure | 1 | 0.03 | | 0.1 | | ns |  | 0.5 | 0.9 | | ns | |  | 0.8 | 1.0 | | ns | |  | 1.1 | 2.3 | | ns |
| Time | 1 | 0.5 | | 0.1 | | ns |  | 9.5 | 15.4 | | *** | |  | 1.4 | 1.8 | | ns | |  | 4.7 | 10.2 | | *** |
| Gr*Ex | 2 | 0.9 | | 0.2 | | ns |  | <0.1 | <0.1 | | ns | |  | 0.5 | 0.6 | | ns | |  | 0.3 | 0.7 | | ns |
| Gr*Ti | 2 | 2.8 | | 0.6 | | ns |  | 0.5 | 0.8 | | ns | |  | 2.1 | 2.8 | | ns | |  | 4.3 | 9.2 | | *** |
| Ex*Ti | 1 | 1.0 | | 0.2 | | ns |  | 0.7 | 1.1 | | ns | |  | 0.1 | 0.1 | | ns | |  | 1.1 | 2.4 | | ns |
| Gr*Ex*Ti | 2 | 0.3 | | 0.1 | | ns |  | 0.3 | 0.5 | |  | |  | 0.2 | 0.2 | | ns | |  | 1.3 | 2.7 | | ns |
| Residuals | 240 | 4.4 | |  | |  |  | 0.6 |  | |  | |  | 0.8 |  | |  | |  | 0.5 |  | |  |
| Total | 251 |  | |  | |  |  |  |  | |  | |  |  |  | |  | |  |  |  | |  |
|  |  |  | |  | |  |  |  |  | |  | |  |  |  | |  | |  |  |  | |  |
| Species |  | *Sparisoma frondosum* | | | | |  | *Stegastes fuscus* | | | | |  | *Chromis multilineata* | | | | |  | *Malacoctenus delalandii* | | | |
| Source | *df* | MS | F | | *P* | |  | MS | | F | | *P* |  | MS | F | | *P* | |  | MS | F | *P* | |
| Groups | 2 | 74.4 | 36.3 | | *** | |  | 154.8 | | 105.0 | | *** |  | 0.6 | 6.6 | | *** | |  | 6.5 | 5.6 | *** | |
| Exposure | 1 | 24.6 | 11.9 | | *** | |  | 63.2 | | 42.9 | | *** |  | 0.6 | 6.9 | | *** | |  | 8.9 | 7.6 | *** | |
| Time | 1 | 9.3 | 4.5 | | * | |  | 24.2 | | 16.4 | | *** |  | <0.1 | <0.1 | | ns | |  | 8.4 | 7.3 | *** | |
| Gr*Ex | 2 | 18.8 | 9.2 | | *** | |  | 34.3 | | 23.2 | | *** |  | 0.7 | 7.1 | | *** | |  | 3.8 | 3.2 | * | |
| Gr*Ti | 2 | 5.2 | 2.5 | | ns | |  | 11.8 | | 8.0 | | *** |  | <0.1 | <0.1 | | ns | |  | 3.2 | 2.8 | * | |
| Ex*Ti | 1 | 5.2 | 2.5 | | ns | |  | 29.3 | | 19.9 | | *** |  | <0.1 | 0.6 | | ns | |  | 0.1 | 0.1 | ns | |
| Gr*Ex*Ti | 2 | 2.9 | 1.4 | | ns | |  | 11.4 | | 7.7 | | *** |  | <0.1 | 0.3 | | ns | |  | 3.6 | 3.1 | ns | |
| Residuals | 240 | 2.0 |  | |  | |  | 1.5 | |  | |  |  | 0.1 |  | |  | |  | 1.1 |  |  | |
| Total | 251 |  |  | |  | |  |  | |  | |  |  |  |  | |  | |  |  |  |  | |
