# Supplementary material for: The Farther the Better: Effects of Multiple Environmental Variables on Reef Fish Assemblages along a Distance Gradient from River Influences
Source: PLoS One. 2016 Dec 1;11(12):e0166679. doi: 10.1371/journal.pone.0166679 (PMC5131968; doi:10.1371/journal.pone.0166679)
Supplement: S6 Table — Predictor variables that influenced significantly descriptors of fish assemblage according to simple regression. r, Pearson correlation coefficient. Levels of significance: ***earP<0.001, ** = P<0.01, * = P<0.05. (DOCX) [file pone.0166679.s007.docx]

**S6 Table.**

| Fish parameter | Distance from the continental influence | |  | Boulder size | |  | Number of refuges | |  | | Benthic cover | |
| --- | --- | --- | --- | --- | --- | --- | --- | --- | --- | --- | --- | --- |
|  | r | p |  | r | p |  | r | p | |  | r | p |
| Richness | 0.7 | *** |  | 0.6 | *** |  | -0.2 | * | |  |  |  |
| Abundance | 0.3 | ** |  | 0.2 | * |  |  |  | |  |  |  |
| Biomass | 0.8 | *** |  | - | - |  |  |  | |  |  |  |
| Trophic group diversity | 0.9 | *** |  | 0.6 | *** |  |  |  | |  | -0.8 | *** |
